# Dynamic Arc SUMOylation and Selective Interaction with F-Actin-Binding Protein Drebrin A in LTP Consolidation *In Vivo*

**DOI:** 10.3389/fnsyn.2017.00008

**Published:** 2017-05-10

**Authors:** Rajeevkumar R. Nair, Sudarshan Patil, Adrian Tiron, Tambudzai Kanhema, Debabrata Panja, Lars Schiro, Kamil Parobczak, Grzegorz Wilczynski, Clive R. Bramham

**Affiliations:** ^1^Department of Biomedicine and KG Jebsen Centre for Neuropsychiatric Disorders, University of BergenBergen, Norway; ^2^Laboratory of Molecular and Systemic Neuromorphology, Department of Neurophysiology, Nencki Institute of Experimental BiologyWarsaw, Poland

**Keywords:** actin cytoskeleton, brain-derived neurotrophic factor (BDNF), dentate gyrus, hippocampus, immediate early protein, long-term potentiation (LTP), small ubiquitin-like modifier (SUMO), synaptic plasticity

## Abstract

Activity-regulatedcytoskeleton-associated protein (Arc) protein is implicated as a master regulator of long-term forms of synaptic plasticity and memory formation, but the mechanisms controlling Arc protein function are little known. Post-translation modification by small ubiquitin-like modifier (SUMO) proteins has emerged as a major mechanism for regulating protein-protein interactions and function. We first show in cell lines that ectopically expressed Arc undergoes mono-SUMOylation. The covalent addition of a single SUMO1 protein was confirmed by *in vitro* SUMOylation of immunoprecipitated Arc. To explore regulation of endogenous Arc during synaptic plasticity, we induced long-term potentiation (LTP) in the dentate gyrus of live anesthetized rats. Using coimmunoprecipitation of native proteins, we show that Arc synthesized during the maintenance phase of LTP undergoes dynamic mono-SUMO1-ylation. Levels of unmodified Arc increase in multiple subcellular fractions (cytosol, membrane, nuclear and cytoskeletal), whereas enhanced Arc SUMOylation was specific to the synaptoneurosomal and the cytoskeletal fractions. Dentate gyrus LTP consolidation requires a period of sustained Arc synthesis driven by brain-derived neurotrophic factor (BDNF) signaling. Local infusion of the BDNF scavenger, TrkB-Fc, during LTP maintenance resulted in rapid reversion of LTP, inhibition of Arc synthesis and loss of enhanced Arc SUMO1ylation. Furthermore, coimmunoprecipitation analysis showed that SUMO1-ylated Arc forms a complex with the F-actin-binding protein drebrin A, a major regulator of cytoskeletal dynamics in dendritic spines. Although Arc also interacted with dynamin 2, calcium/calmodulindependentprotein kinase II-beta (CaMKIIβ), and postsynaptic density protein-95 (PSD-95), these complexes lacked SUMOylated Arc. The results support a model in which newly synthesized Arc is SUMOylated and targeted for actin cytoskeletal regulation during *in vivo* LTP.

## Introduction

Activity-regulated cytoskeleton-associated protein (Arc) has been identified as an indispensable component of multiple forms of protein synthesis-dependent plasticity, including long-term potentiation (LTP), long-term depression (LTD) and related homeostatic synaptic scaling (Bramham et al., 2010; Korb and Finkbeiner, 2011; Shepherd and Bear, 2011). Behaviorally, Arc synthesis is critical to long-term memory formation and processes of extinction and reconsolidation, as well as postnatal development of the visual cortex (Guzowski et al., 2000; Plath et al., 2006; McCurry et al., 2010; Trent et al., 2015). Arc is rapidly transcribed in response to glutamatergic synaptic signaling and transported to dendrites for local translation and synaptic action of the protein; Arc is also synthesized in the cell body and acts in the nucleus (Bloomer et al., 2007; Bramham et al., 2010; Korb et al., 2013; Steward et al., 2015).

In LTD and homeostatic scaling, Arc forms a complex with dynamin 2 and endophilin 3 to facilitate endocytosis of AMPA-type glutamate receptors (Chowdhury et al., 2006; Shepherd et al., 2006; Peebles et al., 2010; DaSilva et al., 2016). During LTP consolidation, newly synthesized Arc promotes stable increases in filamentous (F-) actin implicated in the structural enlargement of dendritic spines (Messaoudi et al., 2007). In inverse synaptic tagging, Arc is targeted to less active synapses through binding to inactive calcium/calmodulin-dependent protein kinase II–beta (CaMKIIβ; Okuno et al., 2012). In the nucleus, Arc interacts with multiple proteins to modulate transcription and chromatin state (Bloomer et al., 2007; Korb et al., 2013; Wee et al., 2014; Oey et al., 2015). Structurally, Arc is a flexible protein comprised of two major domains flanking a central, mostly disordered hinge region (Myrum et al., 2015; Zhang et al., 2015).

The ability of Arc to interact with diverse protein partners may explain its functional versatility. However, the mechanisms that regulate Arc protein localization and protein-protein interactions are little known. The covalent attachment of small ubiquitin-like modifier (SUMO) has emerged as a major mechanism for regulating protein localization, activity, and function and many of these effects are mediated via SUMO-directed protein-protein interactions (Gareau and Lima, 2010; Flotho and Melchior, 2013; Hay, 2013). Four SUMO paralogs (SUMO-1-4) have been identified in mammals, though only SUMO1-3 are ubiquitously expressed. SUMO2 and SUMO3 differ by only 3 amino acids and are collectively referred to as SUMO2/3. In neurons, SUMOylation dependent regulation of numerous proteins including regulators of neurotransmitter release and postsynaptic glutamatergic signaling has been demonstrated (Martin et al., 2007; Kantamneni et al., 2011; Henley et al., 2014; Craig et al., 2015; Schorova and Martin, 2016). Neuronal activity regulates the distribution of the SUMOylation machinery and SUMOylation is required for LTP induction and hippocampal-dependent memory formation (Loriol et al., 2013; Lee et al., 2014).

Arc has recently been identified as an *in vitro* SUMO substrate (Bramham et al., 2010; Craig et al., 2012). However, SUMOylation of endogenous Arc in the context of synaptic plasticity has not been explored. Here, we show that newly synthesized Arc is rapidly SUMOylated during LTP consolidation in the dentate gyrus of live rats. SUMO1 conjugated Arc is concentrated to the synaptic, cytoskeletal fraction where it forms a complex with drebrin A, a regulator of F-actin stability in dendritic spines. Although Arc also interacts with dynamin 2, CaMKIIβ and postsynaptic density protein-95 (PSD-95), these complexes lack SUMOylated Arc. The results support a model in which SUMO1-ylation targets Arc for regulation of actin cytoskeletal dynamics in LTP.

## Materials and Methods

### Materials

Antibodies: Arc C7 mouse monoclonal (1:200, sc-17839), Arc H300 rabbit polyclonal (1:200, sc-15325), Cofilin (1:500, sc-32158), Drebrin A (1:200, #sc-374269), Dynamin 2 (1:1000, sc-6400), GAPDH (1:5000, sc-32233), Histone 1 (1:500, sc-10806), rabbit polyclonal SUMO1 (1:1000, sc-9060), mouse monoclonal SUMO1 (1:1000, sc-5308), SUMO2/3 (1:1000, sc-32873), normal mouse IgG and rabbit IgG were from Santa Cruz Biotechnology. Arc Synaptic Systems (1:1000, 156003) β-actin (1:5000 Sigma, #F3022), Cofilin (1:500, Cell signaling #5175), Drebrin A (1:500, Cell signaling #12243S), CaMKIIα (1:500, Chemicon, #MAB8699), CaMKIIβ (Invitrogen #139800), His6-tag (1:1000, Millipore #5531), PSD-95 (1:1000, Thermo scientific #MA1-045), Vimentin (1:1000, Sigma #V5225).

Recombinant TrkB-Fc (stock 100 μg/ml, #688-TK) and control human IgG-Fc (100 μg/ml, #110-HG) were obtained from R&D Systems and diluted in phosphate-buffered saline (PBS) containing 0.1% bovine serum albumin. The cDNA encoding Arc was a gift from Dr. Joseph Dynes, University of California, Irvine, USA. Expression constructs for His6-tagged SUMO1, 2 and 3 were kindly provided by Dr. Ronald Hay, University of Dundee, UK.

### Generation of Arc Expression Constructs

Arc cDNA (amino acid residues 1-396) was cloned between the *Hind*III and *Bam*HI sites in pCDNA3.1 (+). Restriction and modification enzymes and other molecular biology related chemicals were from New England Biolabs or Fermentas. The sequences of the Arc constructs were confirmed by DNA sequencing by the dideoxy chain termination method in an automated DNA sequencer (ABI Prism 310).

### Expression of Arc in HT-1080 Cells

HT-1080 (Human fibrosarcoma) cells were cultured as a monolayer in DMEM containing 10% FBS and antibiotics (penicillin 100 units/ml, streptomycin 100 μg/ml, fungizone 2.5 μg/ml) at 37°C in a humid atmosphere having 5% CO_2_. For transient transfection, HT-1080 cells were seeded on 35 mm or 100 mm sterile petri dishes. After 24 h, the cells were transfected with plasmid constructs using Lipofectamine 2000 according to the manufacture’s protocol (0.750 μg or 5 μg of plasmid DNA per 35 mm or 100 mm petriplate, respectively). Transfection grade plasmids were prepared using plasmid Midi kit from Qiagen.

### Nickel Affinity Chromatograhpy

Extract from HT-1080 cells coexpressing Arc and His6-tagged SUMO1, 2 or 3 was subjected to nickel affinity chromatography and subsequent immunoblotting analysis was performed using Arc antibody. Ni^+^-nitrilotriacetate (NTA) agarose beads from Qiagen (50 μg/purification) were washed three times using dilution/wash buffer containing NaH_2_PO_4_ (50 mM), NaCl (300 mM) and imidazole (10 mM). Washed beads were incubated overnight at 4°C with approximately 300 μg lysate from transfected cells prepared in RIPA lysis buffer containing 1× protease inhibitor, diluted 10 times with the dilution/wash buffer. The incubated beads were washed three times using dilution/wash buffer and centrifuged at 500× g. Subsequently the bound proteins were eluted in 30 μl elution buffer containing NaH_2_PO_4_ (50 mM), NaCl (300 mM) and imidazole (250 mM). The samples were denatured, resolved by SDS-PAGE, and subjected to immunoblotting.

### *In Vitro* SUMOylation of Immunoprecipitated Arc

HT-1080 cells seeded in 100 mm culture plates were transfected with 5 μg of plasmid, using Lipofectamine 2000. After 24 h from the start of lipofection the cells were lysed either in RIPA lysis buffer (Santacruz sc-24948A) containing 1× protease inhibitor cocktail and 10 mM N-ethylmaleimide (NEM) or a modified RIPA buffer containing 20 mM Tris-HCl (pH 7.4), 150 mM NaCl, 0.5% Triton, 5% glycerol, 10 mM NEM and 1× protease inhibitor cocktail. The homogenate was then centrifuged at 20,000× g for 5 min at 4°C, and the supernatant was collected for subsequent immunoprecipitation. A preliminary Arc-immunoblot was carried out with equal amount of total proteins, to quantitate the amount of overexpressed protein present in the cell lysate. Immunoprecipitation was performed using anti-Arc antibody (C7 Santa Cruz) and the precipitate was used as the substrate for the *in vitro* SUMOylation assay carried out with E1 activating and E2 conjugating enzymes according to the manufacturer’s instructions (SUMOlink SUMO1, Active Motif).

### Animals

*In vivo* electrophysiological experiments were carried out on 105 adult (60–80 day old) male rats of the Sprague-Dawley outbred strain (Taconic Europe, Ejby, Denmark), weighing 250–350 g. Dentate gyrus tissue was also obtained from 10 naïve, anesthetized rats. Rats had free access to food and water and were on a 12-h light/dark cycle. This research is approved by Norwegian National Research Ethics Committee in compliance with EU Directive 2010/63/EU, ARRIVE guidelines. Persons involved in the animal experiments have approved Federation of Laboratory and Animal Science Associations (FELASA) C course certificates and training.

### Electrophysiology and Intrahippocampal Infusion

The electrophysiological methods have been detailed elsewhere (Panja et al., 2009). Briefly, rats were anesthetized with urethane (1.5 g/kg) and electrodes were stereotaxically positioned for unilateral stimulation of the medial perforant path (7.9 posterior to bregma, 4.2 lateral and depth 2.5) and recording of evoked field potentials in the dentate gyrus (3.7 posterior, 2.2 lateral, depth 2.8). Drugs were infused above via a glass micropipette attached to an infusion pump (infusion rate was 0.06 μl/min) connected via a polyethylene (PE50) tube to a 5 μl Hamilton syringe (Reno, NV). The recording electrode and infusion pipette were clamped together on a micromanipulator with a vertical tip separation of 700 μm. The tip of the infusion cannula was located in deep stratum lacunosum-moleculare of field CA1, approximately 300 μm dorsal to the synaptic zone of medial perforant path-granule synapses in the upper blade of the dorsal dentate gyrus. Test pulses were applied at 0.033 Hz throughout the experiment except during the period of HFS. Responses were allowed to stabilize and 20 min of baseline recordings were obtained. HFS was given in three sessions with 5 min between them. Each session consisted of four, 400 Hz stimulus trains (8 pulses/ train) and the interval between trains was 10 s. Total HFS duration was 10.5 min and the total pulse number was 96.

### Tissue Dissection, Homogenization and Immuonprecipitation

At the end of electrophysiological recording, rats were decapitated and the dentate gyri were rapidly dissected on ice and homogenized in buffer containing 20 mM Hepes pH 7.4, 137 mM NaCl, 1 mM EDTA, 1 mM NaF, 1 mM sodium orthovanadate, 0.5% NP-40, 1 mM DTT, 20 mM NEM and protease inhibitor cocktail (Roche, # 11836170001). Homogenization was performed manually with 10–12 gentle strokes in a tissue grinder with a clearance of 0.1–0.15 mm (Thomas Scientific, Swedesboro, NJ, USA). Protein concentration was measured using BCA protein assay (Pierce, # 23227). Homogenates were stored at −80°C until use.

Two micrograms of antibody was incubated for 1 h at room temperature with 20 μl of washed protein G-agarose beads or protein A/G mix magnetic beads (Cat. No. LSKMAGAG02, Millipore) for each immunoprecipitation. Two-hundred to two-hundred and fifty micrograms lysate (from dentate gyrus, synaptoneurosomes, or the cytoskeletal fraction) was incubated with antibody-bound beads at 4°C for 2 h or overnight. Immunoprecipitates were washed four times with washing buffer (20 mM Hepes pH 7.4, 137 mM NaCl, 1 mM EDTA, 20 mM NEM) and proteins were eluted from the beads by boiling in reducing sample buffer. In the analysis of NEM sensitivity, NEM was omitted from the lysis and immunprecipitation buffers.

### *In Vitro* SENP1 Treatment

To assay the hydrolysis activity of SENP1 *in vitro*, immunoprecipitates were washed three times with lysis buffer and beads were divided equally. The washed beads were incubated in 30 μl reaction buffer (150 mM NaCl, 50 mM Tris/HCl and 5 mM dithiothreitol) containing 300 nM of the purified catalytic domain of SENP1 (E-700-050, R&D system or UW9760, Enzo life sciences) for 2 h at 37°C. SENP1 was omitted from control samples. Every 15 min, the Eppendorf tube was hand-shaken to facilitate the reaction. After incubation, the reactions were terminated by adding 2× sample loading buffer and subjected to SDS/PAGE analysis and immunoblotting subsequently. NEM was included in the lysis buffer prior to immunoprecipitation.

### SDS–PAGE and Immunoblotting

Samples were boiled in sample buffer (Bio-Rad) and resolved via 10% or 8% SDS-PAGE mini gels. Proteins were transferred to nitrocellulose membranes (Amersham Biosciences), blocked with 5% non-fat dry milk, probed with horseradish peroxidase-conjugated anti-rabbit or anti-mouse secondary antibodies (1:10,000, Calbiochem) and developed using chemiluminescence reagents (ECL, Pierce). For coimmunoprecipitation analysis of SUMOylated Arc, antibody-treated blots were stripped with 100 mM 2-mercaptoethanol, 2% SDS and 62.5 mM Tris-HCl, pH 6.7 at 50°C for 30 min, washed, blocked and reprobed with antibody recognizing a different protein (Arc or SUMO).

### Synaptoneurosomal Preparation

At the end of electrophysiological recording, rats were decapitated and the dentate gyri (treated and control) were rapidly dissected on ice and homogenized in 5 ml of homogenization buffer (0.32 M sucrose, 1 mM EDTA, 1 mg/ml BSA and 5 mM HEPES pH 7.4) and centrifuged at 3000× g for 10 min at 4°C. The resulting supernatant was centrifuged at 14,000 rpm for 12 min at 4°C and the pellet resuspended in 550 μl Krebs–Ringer buffer (140 mM NaCl, 5 mM KCl, 5 mM glucose, 1 mM EDTA and 10 mM HEPES pH 7.4). To this, 450 μl of Percoll (45% v/v) was added and mixed, and a synaptoneurosome-enriched top layer was collected after centrifugation at 14,000 rpm for 2 min at 4°C. The fraction was washed and resuspended in 400 μl HEPES-Krebs solution (140 mM NaCl, 3 mM KCl, 10 mM glucose, 2 mM MgSO_4_, 2 mM CaCl_2_ and 10 mM HEPES pH 7.4) equilibrated at 37°C for 10 min. Isolated synaptoneurosomes were lysed in 20 mM Tris-HCl (pH 7.4), 150 mM NaCl, 0.5% Triton, 5% glycerol, 10 mM NEM and 1× protease inhibitor cocktail and used for biochemical analysis.

### Subcellular Fractionation

Rat dentate gyrus tissue was excised, weighed and rinsed in PBS (pH 7.4). A Subcellular Protein Fractionation Kit for Tissues (Thermo Scientific # 87790) was used to separate cytoplasmic, membrane, nuclear soluble, chromatin-bound nuclear and cytoskeletal protein extracts, according to the manufacturer’s instructions. The tissue was first homogenized in the Cytoplasmic Extraction Buffer (CEB) using a tissue grinder. The homogenate was transferred to a Thermo Scientific Pierce Tissue Strainer in a 15 ml conical tube and centrifuged for 5 min at 500× g. The strainer with debris was discarded, and the supernatant (cytoplasmic extract) was recovered. The remaining pellet was resuspended in Membrane Extraction Buffer (MEB) and incubated at 4°C for 10 min with gentle mixing. The membrane extract was recovered by centrifugation at 3000× g for 5 min. The pellet was then resuspended in Nuclear Extraction Buffer (NEB) and incubated at 4°C for 30 min with gentle mixing. The soluble nuclear extract was separated by centrifugation at 5000× g for 5 min. NEB containing micrococcal nuclease was added to the pellet and incubated at 37°C for 15 min with gentle mixing. Chromatin-bound nuclear proteins were released and recovered by centrifugation of 16,000× g for 5 min. The remaining pellet was resuspended in Pellet Extraction Buffer (PEB) and incubated at room temperature for 10 min. The cytoskeletal extract was recovered by centrifugation at 16,000× g for 5 min.

### Densitometry and Statistical Analylsis

Immunoblots were scanned using Gel DOC EQ (BIO RAD) and band intensities were quantified using analytical software (Quantity one 1D analysis software, BIORAD, Hercules, CA, USA).

Densitometric values from the treated dentate gyrus (+) were expressed as fold change relative to contralateral control dentate gyrus (−). For analysis of SUMOylation state, intensity of upper 65 kDa band (immunoreactive for Arc and SUMO1) was normalized to 50 kDa non-modifed Arc. Pairwise comparisons of means were evaluated with a two-tailed Student’s *t*-test using Graphpad prism software. The *p*-value for significance was 0.05.

## Results

### SUMOylation of Ectopically Expressed Arc and *In Vitro* SUMOylation Assay

**Figure 1:**
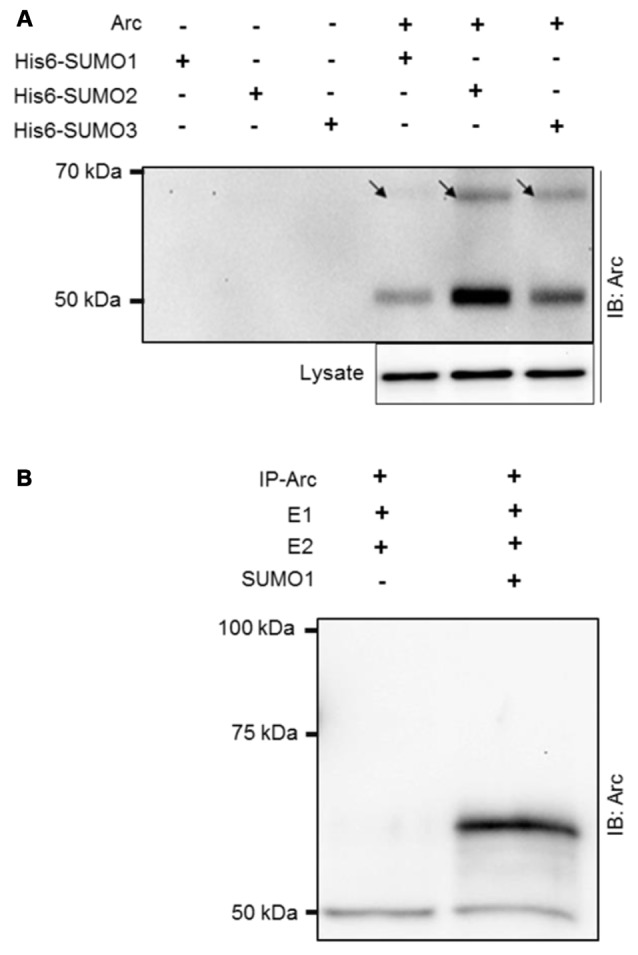
**SUMOylation of ectopically expressed activity-regulatedcytoskeleton-associated protein (Arc) and *in vitro* SUMOylation assay. (A)** Ectopically expressed Arc is SUMOylated and forms non-covalent complexes with small ubiquitin-like modifier 1 (SUMO1) and SUMO2/3 moieties in HT-1080 fibrosarcoma cell lines. Extract of cells coexpressing Arc with His6-tagged SUMO 1, 2 or 3 were subjected to nickel affinity chromatography and immunoblotted for Arc. A high molecular mass band at ~65 kDa (arrow) corresponding to the covalent addition of single His6-tagged SUMO1, SUMO2, or SUMO3 to Arc was detected. The molecular mass of His6-SUMOylated Arc is higher than physiologically SUMOylated Arc, due to histidine tag residues. The presence of non-modified Arc protein indicates non-covalent complex formation of Arc with His-SUMO modified proteins or free SUMO. **(B)** Arc was immunoprecipitated from HT-1080 cells expressing non-tagged Arc protein and *in vitro* SUMOylation was performed with SUMO activating (E1) and conjugating (E2) enzymes in the presence or absence of SUMO1. Upon addition of SUMO1, an Arc immunoreactive band at 65 kDa was detected, corresponding to single SUMO1ylation of Arc. Equal amounts of protein were loaded. No bands were observed in the gel above the 100 kDa marker.

Arc was ectopically expressed in HT-1080 fibrosarcoma cells together with His6-tagged SUMO1, SUMO2, or SUMO3. His-associated protein complexes were purified by nickel affinity chromatography and immunoblotted for Arc (Figure 1A). A high molecular mass band at ~65 kDa corresponding to the covalent addition of single, His6-tagged SUMO to Arc was detected for all three SUMO isoforms, with strongest bands detected for SUMO2 and SUMO3 in this context (Figure 1A). Furthermore, the detection of non-modified Arc (50 kDa) in the His-SUMO pulldown indicated that Arc also interacts non-covalently with SUMOylated proteins or free SUMO. Covalent SUMO modification was demonstrated in an vitro SUMOylation assay using immunoprecipitated Arc as a substrate for a conjugation reaction mediated by E1 and E2 enzymes. Upon addition of SUMO1 to the reaction, an Arc immunoreactive band at 65 kDa corresponding to the conjugation of the 11 kDa SUMO1 protein to native Arc was detected (Figure 1B).

### SUMOylation of Endogenous Arc Following LTP Induction in the Dentate Gyrus *In Vivo*

**Figure 2:**
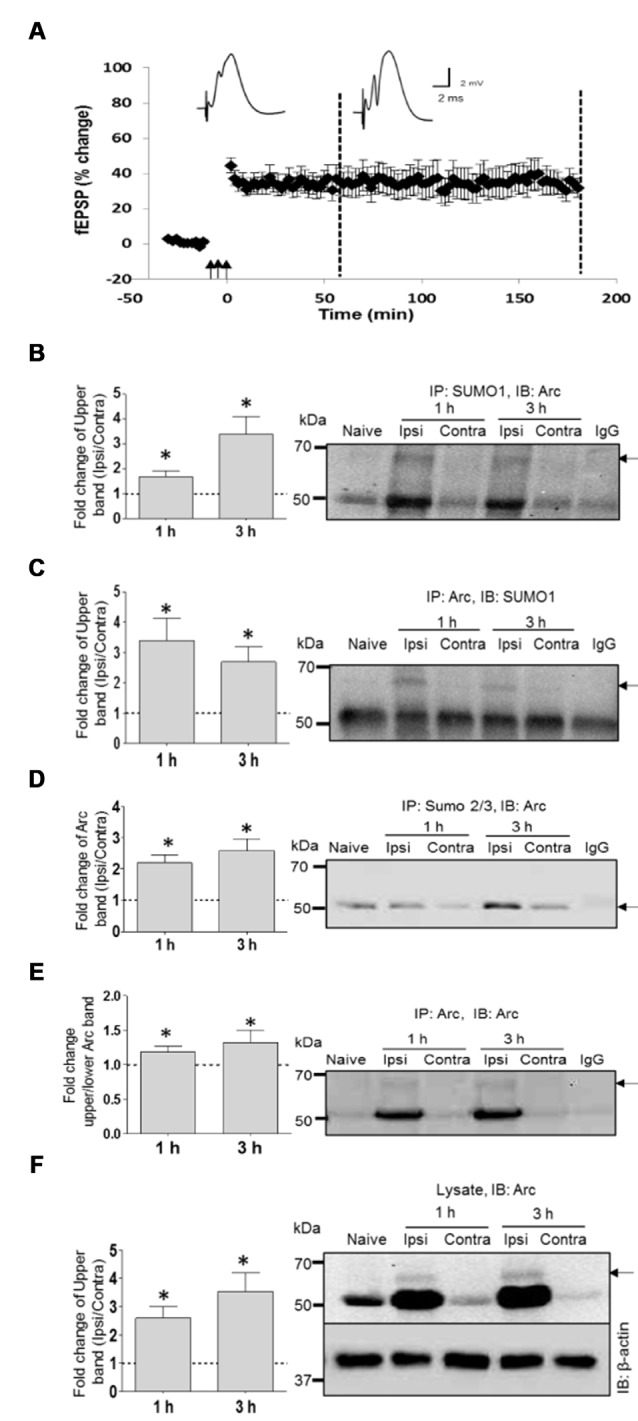
**SUMO1ylation of endogenous Arc following *in vivo* long-term potentiation (LTP) induction. (A)** Time course plots of medial perforant path-dentate gyrus evoked field excitatory postsynaptic potential (fEPSP) recorded before and after high-frequency stimulation (HFS; indicated by arrows). Values are mean ± SEM of the maximum fEPSP slope expressed in percent of baseline. Test pulses were applied at a 0.033 Hz. HFS (3 × 400 Hz bursts) as indicated by the arrows. Rats were killed at 1 h and 3 h post-HFS (stippled line) and dentate gyrus was microdissected for biochemical analysis. **(B–F)** Coimmunoprecipitation analysis of dentate gyrus extracts. Bar graphs (left panels) based on densitometric analysis of indicated bands expressed as fold change in the ipsilateral HFS-treated dentate gyrus relative to the contralateral, non-stimulated side. Right panels show representative immunoblots from LTP experiments and naïve dentate gyrus. IgG lane is IgG-coupled beads plus lysate from HFS-treated dentate gyrus. **(B)** SUMO1 immunoprecipitation (rabbit polyclonal) followed by immunoblotting with Arc C7 antibody. Quantification of upper band (SUMOylated Arc). **(C)** Arc immunoprecipitation followed by SUMO1 immunoblot. Quantification of upper band (SUMO-Arc). The band at 50 kDa is non-specific (IgG). **(D)** SUMO2/3 immunoprecipitation followed by Arc immunoblot. **(E)** Arc immunoprecipitation (H300) followed by Arc (C7) immunoblot. Fold change in Arc SUMOylated state based on upper/lower Arc band intensity. **(F)** Arc immunoblot in dentate gyrus lysate input samples. β-Actin was used as a loading control. *n* = 4/5; Student’s *t*-test, *P < 0.05.

SUMOylation of ectopically expressed Arc may be unphysiological, as shown for many *in vitro* SUMO substrates (Tatham et al., 2009). We used coimmunoprecipitation to assess SUMOylation of endogenous Arc under basal (non-stimulated) conditions and after induction of LTP in the dentate gyrus of anesthestized rats. Stable field excitatory postsynaptic potential (fEPSP) LTP was induced by brief bursts of high-frequency stimulation (HFS) applied to the medial perforant path input to the dentate gyrus of one hemisphere, while the contralateral dentate gyrus served as a non-stimulated, internal control (Figure 2A). Immunoprecipitation using a SUMO1-specific antibody followed by Arc immunoblotting demonstrated a high molecular weight band at 65 kDa corresponding to single, SUMO1-ylated Arc (Figure 2B), as seen in the *in vitro* SUMOylation assay (Figure 2B; Supplementary Figure S1A). Conversely, a 65 kDa SUMO1-immunoreactive band was detected following immunoprecipitation of Arc (Figure 2C, Supplementary Figure S1B). Levels of SUMOylated Arc (upper band quantification) were low in non-stimulated dentate gyrus from the contralateral hemisphere or naïve anesthetized rats, and increased 2–3 fold post-HFS (Figure 2B). No heavy Arc immunoreactive band was detected following SUMO2/3 immunprecipitation (Figure 2D), indicating that endogenous Arc is selectively modified by SUMO1. Unmodified 50 kDa Arc was prominent in both the SUMO1 and SUMO2/3 pellet from HFS-treated tissue but not in lysates incubated in control IgG-coated beads (Figures 2B,D), indicating non-covalent interaction of Arc with SUMOylated proteins.

SUMOylation could be mediated by constitutive activity of the SUMOylation machinery, or enhanced activity resulting in a higher proportion of SUMOylated to unmodified Arc protein (enhanced SUMOylation state). To estimate changes in Arc SUMOylation state in LTP, we immunoprecipitated Arc and measured the ratio of upper band (SUMO-Arc) to lower band intensity in the Arc immunoblot (Figure 2E). Arc SUMOylation was significantly enhanced in the HFS treated dentate gyrus relative to control at 1 and 3 h post-HFS.

**Figure 3:**
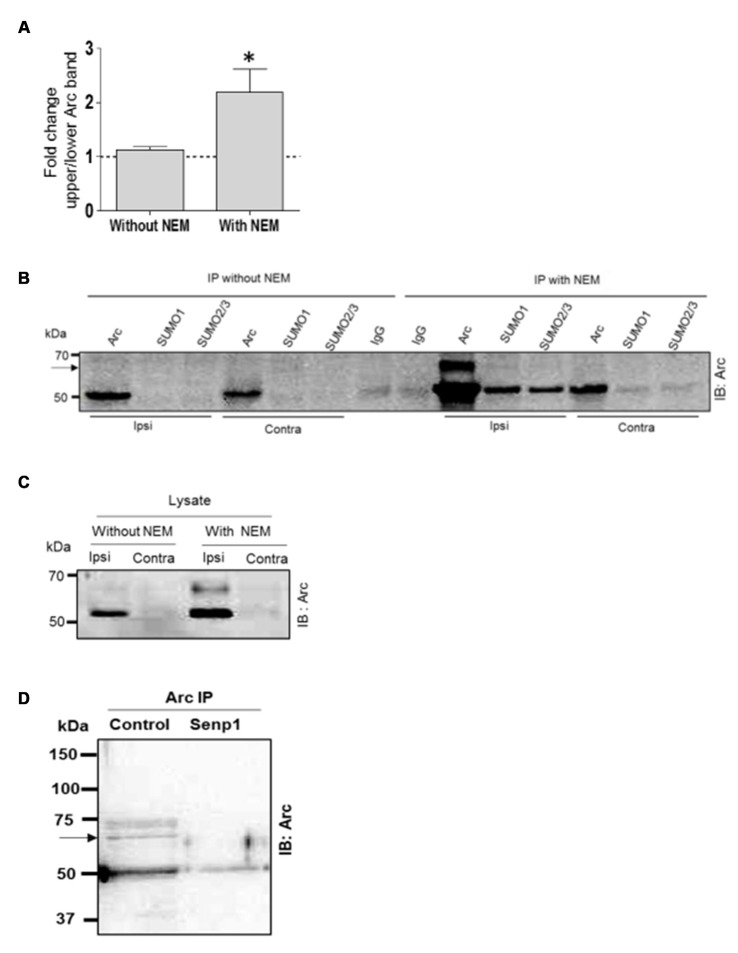
**N-ethylmaleimide (NEM) dependent retention of SUMO-modified Arc.** Coimmunoprecipitation was carried with and without the addition of the cysteine (SUMO) protease inhibitor NEM to the lysis and immunoprecipitation buffer. **(A)** Detection of enhanced Arc SUMOylation during LTP is NEM-dependent. The ratio of upper to lower Arc immunoreacitive bands was determined in Arc immunoprecipitates. Bar graphs show fold change in HFS-treated dentate gyrus compared to control. *n* = 5; Student’s *t*-test, *P < 0.05. **(B)** Immunoprecipitation of Arc, SUMO1 and SUMO2/3 was performed in the presence or absence of NEM and precipitates were immunoblotted for Arc. Representative blot from a single gel. A 65 kDa immunoreactive Arc band (arrow) was reliably detected only in NEM-processed samples. The non-covalent interaction of Arc with SUMO1 and SUMO2/3 was also NEM-dependent. **(C)** Arc immunoblots of dentate gyrus lysate (input) samples. NEM treatment tended to increase Arc immunoreactivity but there was no difference in fold increases in Arc with and without NEM (*n* = 5, *P* > 0.05). **(D)** SENP1 enzymatic treatment of Arc immunoprecipitate abolished 65 kDa SUMO-Arc. In this immuno-precipitation performed with a polyclonal antibody from Synaptic Systems, an additional 75 kDa Arc immunoreactive band was detected by the monoclonal mouse Arc C7 antibody. However, the 75 kDa band could not be validated in the reverse immunoprecipitation or by SUMO1 immunoprecipitation.

SUMO modifications are typically unstable due to activity of SUMO-specific proteases (SENPs). Detection of SUMOylated proteins often requires the addition of the cysteine (and SUMO) protease inhibitor NEM to the lysis. The biochemical experiments in Figure 2 were all performed with NEM added to the lysis and immunoprecipitation buffers. To further validate Arc SUMOylation, assays were performed with and without NEM in the buffers. In the Arc immunoprecipitation analysis shown in Figure 3A, HFS induced a significant 2.2-fold increase in Arc SUMOylation (ratio of upper to lower Arc immunoreactive band) in NEM-treated samples, whereas no change in SUMO-Arc was detected in the absence of NEM (Figures 3A–C). In the SUMO1 precipitate, 65 kDa SUMO-Arc and unmodified Arc were only reliably detected in NEM-treated samples (Figure 3B). The NEM-sensitivity of unmodifed Arc again suggests extensive non-covalent interaction of Arc with proteins that were precipitated by anti-SUMO1 antibody. As an additional validation, we performed a SENP1 enzymatic digestion of Arc immunoprecipitates. SENP1 treatment abolished the 65 kDa SUMO-Arc immunoreactive band present in control samples (Figure 3D). Thus, the coimmunoprecipitation analysis combined with the effects of NEM and SENP1 treatment support *in vivo* SUMOylation of Arc protein in LTP.

### Newly Synthesized, BDNF-Induced Arc Is SUMOylated during LTP Consolidation *In Vivo*

LTP consolidation requires a period of sustained Arc synthesis mediated by persistent BDNF-TrkB activation of Arc translation (Messaoudi et al., 2007; Panja et al., 2009, 2014; Panja and Bramham, 2014). Acute inhibition of Arc translation with antisense RNA or local infusion of the BDNF-scavenger TrkB-Fc results in rapid (minutes) loss of Arc protein and inhibition of LTP maintenance. This suggested that Arc protein involved in LTP is synthesized and degraded in rapid cycles. We therefore considered that Arc SUMOylation could occur on: (1) newly synthesized, labile Arc protein involved in LTP maintenance; (2) newly synthesized, stable Arc protein; or (3) pre-existing Arc protein.

**Figure 4:**
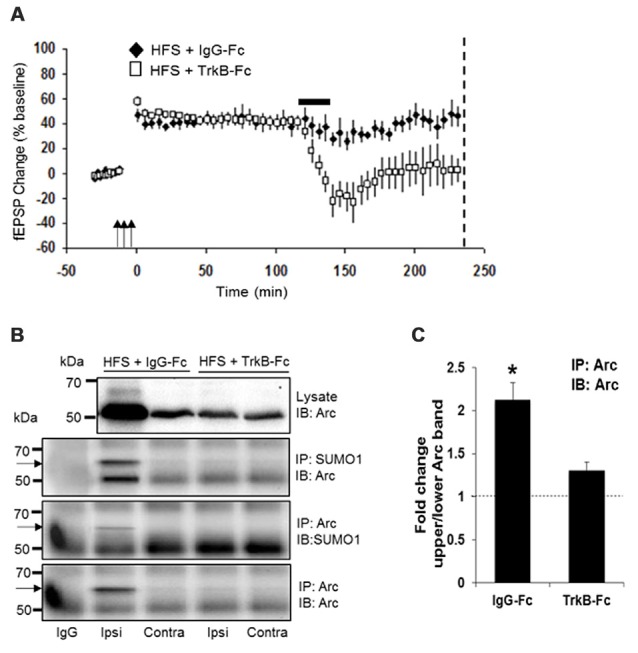
**Newly synthesized brain-derived neurotrophic factor (BDNF)-induced Arc is SUMOylated during LTP consolidation. (A)** Time course plots of medial perforant path-dentate gyrus evoked fEPSPs recorded before and after HFS (indicated by arrows). Values are mean ± SEM of the maximum fEPSP slope expressed as percent of baseline. TrkB-Fc or control IgG-Fc (1 μl, 12.5 min, 100 μg) was infused into the dorsal dentate gyrus at 2 h post-HFS (indicated by a bar) and dentate gyrus tissue was collected at 4 h post-HFS (stippled line). TrkB-Fc infusion reverted ongoing LTP maintenance. **(B)** Bi-directional coimmunoprecipitation of SUMOylated Arc using anti-SUMO1 and anti-Arc antibodies. Enhanced Arc expression and Arc SUMO1ylation in HFS-treated dentate gyrus was reverted to baseline levels by TrkB-Fc infusion. **(C)** Bar graph shows fold change in Arc SUMOylation state in HFS-treated dentate gyrus compared to contralateral control in TrkB-Fc and IgG-Fc infused rats. Arc immunoprecipitation was performed and the ratio of 65 kDa (SUMO-Arc) to unmodified 50 kDa Arc was calculated. *n* = 5; Student’s *t*-test, *P < 0.05.

Two hours after HFS, rats received a unilateral infusion (1 μl, 12.5 min) of TrkB-Fc or control human IgG into the dorsal hippocampus, and dentate gyrus tissue was collected at 4 h post-HFS. As expected, TrkB-Fc infusion resulted in complete reversion of LTP (Figure 4A) and inhibition of Arc expression relative to the IgG-Fc infused control. Coimmuno-precipitation analysis further showed reduction of SUMO1-ylated Arc to controls levels following TrkB-Fc treatment (Figure 4B). Arc SUMOylation state was again estimated by the ratio of upper to lower band intensity in the Arc immunoprecipitate (Figure 4B). A significant 2.1-fold increase in Arc SUMOylation state was abolished by TrkB-Fc treatment (Figure 4C). We conclude that newly synthesized, BDNF-induced Arc is dynamically SUMOylated in the context of LTP consolidation.

### Enhanced Arc SUMOylation in the Synaptoneurosome Compartment

Arc protein exhibits a widespread somatodendritic pattern of expression in dentate granule cells following LTP-induction (Messaoudi et al., 2007; Steward et al., 2015). Arc protein expression is transiently enriched in dendritic spines of medial perforant path synapses (Moga et al., 2004; Rodríguez et al., 2005; Farris et al., 2014; Steward et al., 2015). Functionally, Arc is critical for stable F-actin increases (Messaoudi et al., 2007), and F-actin expansion is essential for long-term enlargement of dendritic spines (Fukazawa et al., 2003; Bramham, 2008; Tanaka et al., 2008; Bosch et al., 2014; Bailey et al., 2015). Arc also accumulates in neuronal nuclei, where it may function in homeostatic plasticity (Korb et al., 2013). If SUMOylated Arc functions in dendritic spines during LTP, it should be present in the glutamatergic synaptic compartment.

**Figure 5:**
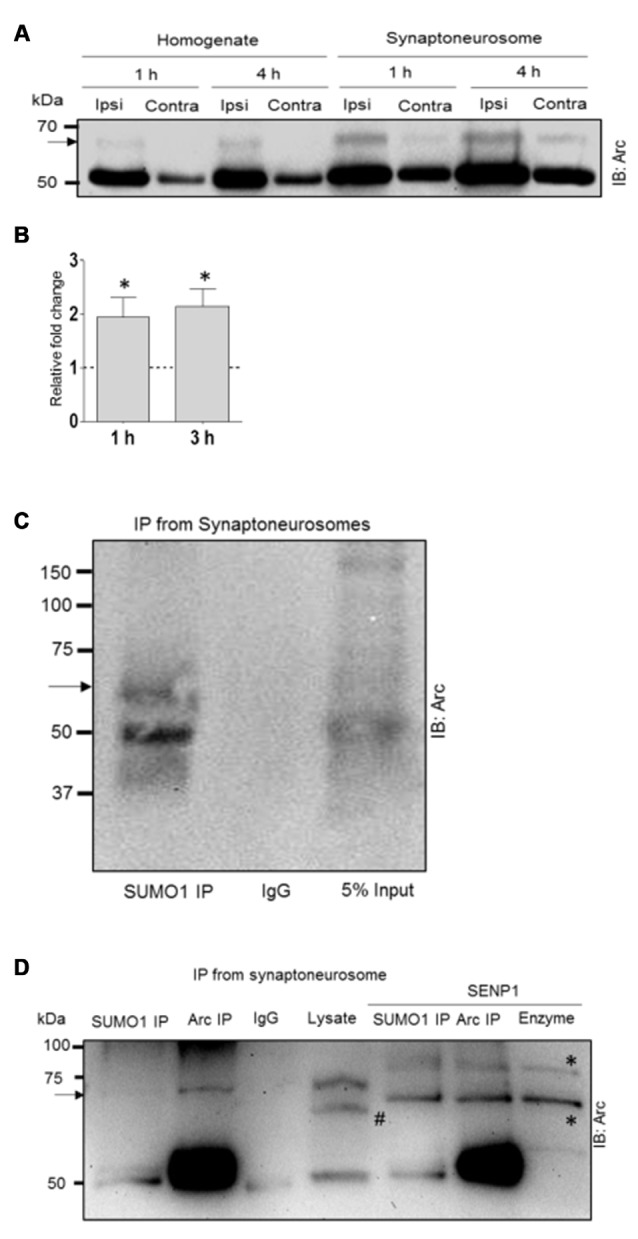
**Enhanced Arc SUMOylation in the synaptoneurosome compartment during LTP *in vivo*. (A)** Representative Arc immunoblot performed in dentate gyrus whole lysate and fractionated synaptoneurosome samples. **(B)** Fold change in SUMOylated Arc based on the ratio of the upper and lower Arc immunoreactive bands. Two dentate gyri were pooled each experiment. *n* = 4; Student’s *t*-test, *P < 0.05. **(C)** SUMO1immuno-precipitation (mouse monoclonal) followed by Arc immunoblotting confirmed Arc SUMOylation in synaptoneurosomes. Unmodified Arc is also detected in the SUMO1 pellet. **(D)** SENP1 digestion removed SUMOylated Arc. The heavy band detected in the Arc and SUMO1 immunoprecipitate is absent in SENP1 treated samples. Arc was immunoprecipitated with mouse monoclonal Arc C7 antibody. ^#^The polyclonal rabbit Arc antibody (Synaptic Systems) used for immunoblotting detects a spurious band in the lysate that is absent in the Arc pellet. *Bands due to SENP1 enzyme in the blot; right lane shows SENP1 enzyme loaded alone. 6% polyacrylamide gel.

To address this question, we examined Arc SUMOylation in fractionated dentate gyrus synaptoneurosomes collected 1, 3 and 4 h after LTP induction. Synaptoneurosomes are highly enriched in pinched-off dendritic spines and well-suited for capturing signaling events in the postsynaptic compartment (Håvik et al., 2003; Kanhema et al., 2006; Panja et al., 2014). Arc protein was enriched in synaptoneurosomes relative to whole dentate lysates in control and HFS-treated dentate gyrus. In non-stimulated dentate gyrus, a 65 kDa Arc-immunoreactive band was detected in synaptoneurosomes but not whole lysate samples (Figure 5A). The heavy 65 kD Arc band was clearly detected in both lysates synaptoneurosomes following HFS-induced upregulation of Arc. Based on the ratio of the upper to lower Arc band intensity, synaptoneurosomes exhibited a significant ~2-fold increase in Arc SUMOylation at 1 and 3 h post HFS (Figure 5B). Immunoprecipitation with anti-SUMO1 antibody performed in synaptoneurosomes from naive dentate gyrus confirmed the 65 kDa band as SUMO1-ylated Arc (Figure 5C). As in whole lysates samples, a single prominent high molecular weight Arc species is observed in the SUMO1 preciptate. Non-covalent coupling to SUMO1ylated proteins is also clearly present in the synaptoneurosomal fraction (Figure 5C). Finally, SENP1 treatment removed the SUMO1-Arc band detected by Arc and SUMO1 immunoprecipitation (Figure 5D). Thus, SUMOylated Arc is enriched in the synaptic compartment under basal conditions and undergoes enhanced expression following LTP induction.

### Enhanced Arc SUMOylation in the Dentate Gyrus Cytoskeletal Fraction

**Figure 6:**
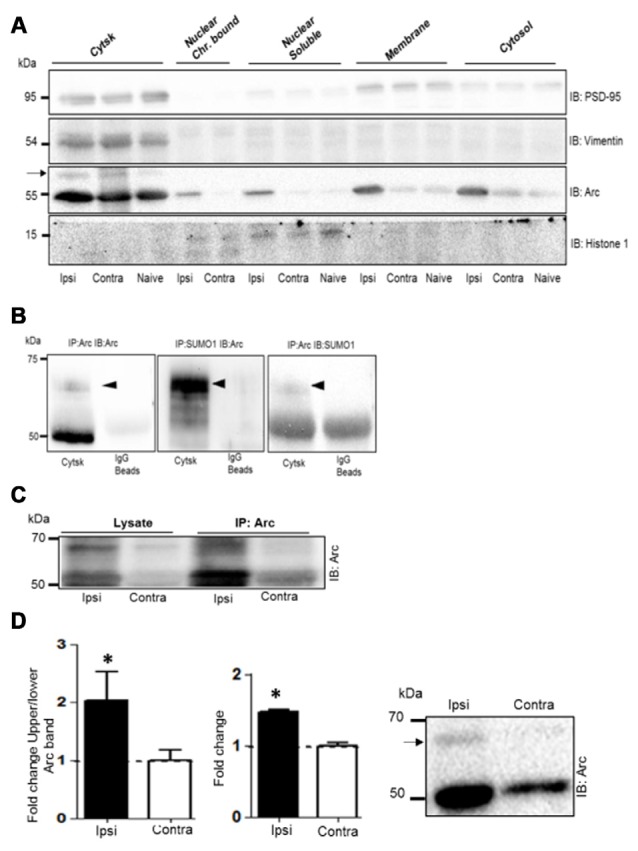
**Enhanced Arc SUMOylation in the cytoskeletal fraction during LTP *in vivo*. (A)** Immunoblot characterization of dentate gyrus subcellular fractions. Ipsilateral HFS-treated (Ipsi), contralateral control (Contra), and Naïve. Vimentin and histone 1 were used as markers of the cytoskeletal and nuclear fractions, respectively. Tissue was collected 1 h post-HFS and fractions collected from four dentate gyri were pooled. A 65 kDa Arc immunoreactive band (indicated by an arrow) was detected in the cytoskeletal fraction. **(B)** Bidirectional co-immunoprecipitation using anti-SUMO1 and anti-Arc antibodies was performed in dentate gyrus cytoskeletal fractions. SUMO1ylated Arc was detected at 65 kDa. Representative blots based on three independent biological replicates. Sameples from two dentate gyrus were pooled. Note the absence of unmodified Arc in the SUMO1 pellet. **(C)** Arc immunoprecipitation was performed in the cytoskeletal fraction from HFS-treated and control dentate gyrus. **(D)** Arc immunoblot analysis of cytoskeletal fraction. Bar graphs shows increase in Arc expression and enhanced Arc SUMOylation based on the ratio of upper/lower Arc bands in the dentate gyrus cytoskeletal fraction at 1 h post-HFS relative to contralateral control. *n* = 5; Student’s *t*-test, *P < 0.05. Representative Arc immunoblots on right. Arrow indicates 65 kDa SUMOylated Arc.

Next we used subcellular fractionation to further identify the subcompartments of for Arc SUMOylation and non-covalent interactions. Five fractions (cytosol, membrane, soluble nuclear, chromatin bound nuclear and cytoskeletal) from dentate gyrus were characterized by immunoblotting using compartment-enriched marker proteins (Figure 6A). Arc immunoblotting revealed expression of the SUMOylated and unmodified Arc in the cytoskeletal fraction of HFS-treated and non-stimulated dentate gyrus (Figure 6A). Following LTP induction, 50 kDa Arc was prominently upregulated in all subcellular fractions (Figure 6A). However, SUMOylated Arc could only be detected in the cytoskeletal fraction. Bi-directional coimmunoprecipitation analysis of pooled denate gyrus cytoskeletal fractions confirmed the 65 kDa band as SUMO1ylated Arc (Figure 6B), and its enhanced expression in HFS-treated dentate gyrus (Figure 6C). In contrast to synaptoneurosomes, non-covalent interaction of Arc with SUMO precipitated proteins was absent in the cytoskeletal fraction (Figure 6C). A quantitative analysis of Arc SUMOylation in the cytoskeletal fraction was by done by Arc immunoblot analysis. As shown in Figure 6D, Arc SUMOylation was significantly enhanced 2.1–fold in the cytoskeletal fraction of HFS-treated dentate gyrus relative to control.

### SUMO1-ylated Arc Interacts with the F-Actin-Binding Protein Drebrin A

Biochemically, Arc is flexible and has numerous binding partners indicating a hub-like function of the protein (Myrum et al., 2015). A major outstanding question is whether Arc protein engages functionally distinct protein partners following LTP-induction. If so, Arc SUMOylation might serve to direct the formation of specific protein-protein interactions within neuronal subcompartments. As SUMOylation was enriched at synapses and not detected in the nuclear fractions, we focused on a set of known Arc interaction partners (CaMKIIα, CaMKIIβ, dynamin 2 and PSD-95) found in postsynaptic dendrites and spines. Arc binding to CaMKIIβ recruits Arc for inverse synaptic tagging (Okuno et al., 2012), binding to dynamin 2 promotes AMPA receptor endocytosis (Chowdhury et al., 2006; Shepherd et al., 2006), while binding to PSD-95 curtails TrkB-coupled phospholipase C signaling (Husi et al., 2000; Cao et al., 2013). Arc immunoprecipitation followed by immunoblotting confirmed interaction with all binding partners in dentate gyrus lysates and suggest an enhanced interaction of Arc with CaMKIIα, CaMKIIβ and dynamin 2 after LTP induction (Supplementary Figures S2A–C). Next, the respective binding partners were immunoprecipitated from HFS-treated dentate gyrus and probed for Arc. The protein interaction complexes formed by CaMKIIα, CaMKIIβ, PSD-95 and dynamin 2 all contained non-modified Arc, but none of these complexes contained detectable levels 65 kDa SUMOylated Arc (Supplementary Figures S2D–F).

**Figure 7:**
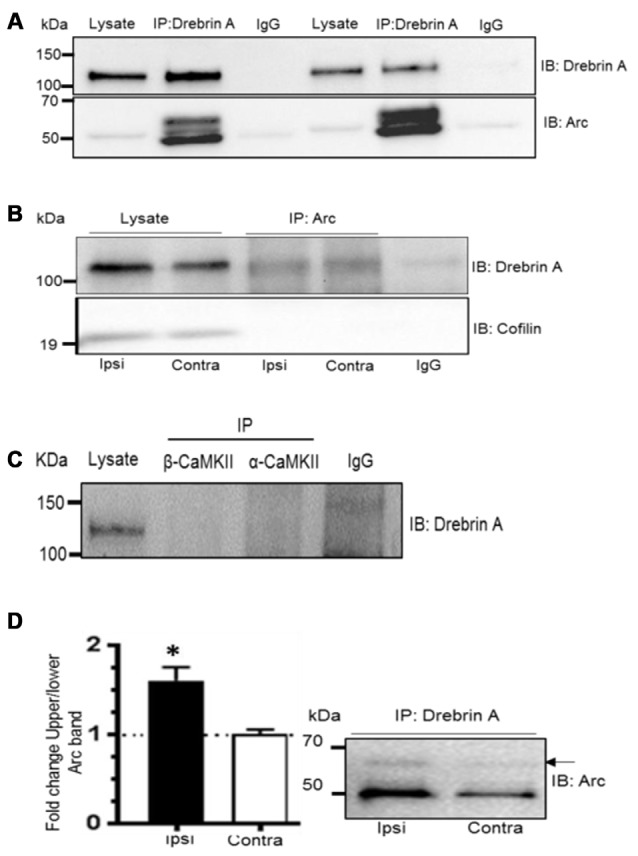
**Enhanced Arc SUMOylation and selective interaction with F-actin-binding protein drebrin A during LTP** *in vivo***. (A)** Arc was detected in complex with drebrin A immunoprecipitated from the dentate gyrus lysates following LTP induction 10% polyacrylamide gel. **(B)** Drebrin A, but not cofilin, was detected in the Arc immunoprecipitate from the cytoskeletal fraction. **(C)** Arc, but not drebrin A, was detected following immunopreciptation of calcium/calmodulindependentprotein kinase II-alpha (CaMKIIα) and CaMKIIβ. Also see Supplementary Figure 2. **(D)** Drebrin A was immunoprecipitated from pooled cytoskeletal fractions and immunoblotted for Arc. Changes in Arc SUMOylation were quantified as the ratio of the upper to lower Arc immunoreactive bands. Arc SUMOylation in complex with debrin A in the cytoskeletal fraction was increased in HFS-treated dentate gyrus relative to control. *n* = 4; Student’s *t*-test, *P < 0.05.

Arc cosediments with a crude F-actin but not with more purified actin preparations suggesting an indirect association of Arc with actin filaments (Lyford et al., 1995). We hypothesized that SUMOylation might target Arc to an F-actin binding protein involved in actin cytoskeletal remodeling in LTP. Coimmunoprecipitation assays were performed with drebrin A and cofilin 1. These actin side-binding proteins are important for the structural and functional plasticity of dendritic spines (Fukazawa et al., 2003; Meng et al., 2005; Messaoudi et al., 2007; Flynn et al., 2012; Bosch et al., 2014; Kojima et al., 2016). Arc and drebrin A were reliably co-precipitated using anti-Arc or anti-drebrin A antibodies for affinity purification (Figures 7A,B), and SUMOylated Arc was clearly detected in the drebrin A precipitate (Figure 7A). In contrast, Arc and cofilin did not coimmunoprecipitate (Figure 7B).

Like drebrin A, CaMKIIα and CaMKIIβ are Arc binding partners that are enriched in the postsynaptic density and cytoskeletal fraction. However, these complexes are distinct as immunoprecipitation of CaMKIIα and CaMKIIβ resulted in copurification of Arc, but not drebrin A (Figure 7C and Supplementary Figure S2). Finally, we examined changes in SUMOylated Arc in complex with cytoskeletal drebrin A during *in vivo* LTP. Drebrin A was immunoprecipitated from pooled dentate gyrus cytoskeletal fractions and probed for SUMOylated Arc. Arc SUMOylation state was significantly enhanced in the drebrin A pellet from HFS-treated dentate gyrus relative to control (Figure 7D). Thus, Arc is SUMOylated during LTP and associates with the synaptic cytoskeletal fraction and the actin regulatory protein drebrin A.

## Discussion

Arc is an indispensable component of both LTP and depression of synaptic transmission, but the mechanisms dictating Arc protein localization and function are not understood. The present study demonstrates SUMOylation of newly synthesized Arc during the maintenance phase of LTP in live anesthetized rats. Arc is conjugated by a single SUMO1 protein under these *in vivo* conditions; no evidence was obtained for polySUMOylation or modification by SUMO2/3. Following LTP induction, BDNF signaling drives synthesis of Arc, which undergoes rapid SUMOylation and association with drebrin A, a regulator of F-actin stability in dendritic spines. Although Arc also coimmunoprecipitates with dynamin 2, CaMKIIβ and PSD-95, these complexes do not contain SUMOylated Arc. Previous work showed that Arc is required for stabilization of F-actin during LTP in the dentate gyrus. The presents work shows that Arc interacts with functionally diverse protein partners following its induction by high-frequency synaptic activation, and specifically links SUMO-Arc to an actin cytoskeletal function of the protein.

Non-modified Arc was massively increased in the nuclear, cytosolic and membrane fractions following LTP induction, but these fractions lacked SUMOylated Arc. Only the synaptoneurosomal and cytoskeletal fractions expressed non-modified and SUMOylated Arc in the basal state and enhanced expression of both following LTP induction. Previous work showed that Arc protein is persistently synthesized and rapidly degraded in LTP consolidation, indicating a critical role for fast-acting Arc protein (Messaoudi et al., 2007; Panja et al., 2009, 2014). Here we found that newly synthesized Arc is rapidly SUMOylated and degraded during LTP.

*In vivo* Arc SUMOylation is shown by coimmunoprecipitation analysis and the sensitivity of SUMO1-Arc to NEM and SENP1 treatment. Changes in SUMOylation state were assessed as the ratio between 65 kDa (SUMO1ylated Arc) to unmodified Arc in the Arc immunoprecipitate. In dentate gyrus lysate samples, synaptoneurosomes, the cytoskeletal fraction, and drebrin A immunoprecipitate, significantly enhanced Arc SUMOylation was found during the LTP maintenance phase. Local inhibition of TrkB signaling reverted Arc synthesis and enhanced SUMOylation, demonstrating dynamic SUMOylation of newly synthesized Arc. Further studies employing SUMO1-Arc specific antibodies or mass spectroscopy are need to definitively quantify changes in Arc SUMOylation state. Recent works suggests that postsynaptic SUMOylation is at least in part regulated by activity-dependent diffusion and transient synaptic trapping of the SUMO conjugating enzyme, Ubc9 (Loriol et al., 2013; Schorova and Martin, 2016).

In the process of inverse synaptic tagging, Arc localizes to less active synapses by binding to inactive CaMKIIβ and further promotes the selective weakening of these synapses by association with dynamin 2 and facilitation of AMPA receptor endocytosis (Okuno et al., 2012). The interaction of Arc with CaMKIIβ and dynamin 2 following LTP induction supports the view that inverse tagging and LTP consolidation are concurrent processes. Although drebrin A, CaMKIIα, CaMKIIβ, PSD-95 are all highly enriched in the postsynaptic compartment and cytoskeletal fraction, SUMO1-Arc was detected only in complex with drebrin A. Taken together this suggests that SUMOylation targets newly synthesized Arc for regulation of actin cytoskeletal dynamics in LTP.

Drebrin A is known to stabilize the suprastructure of the actin cytoskeleton by bundling F-actin or linking F-actin to the PSD (Hayashi et al., 1996; Ivanov et al., 2009; Sharma et al., 2011; Ferhat, 2012; Mikati et al., 2013). Following dentate gyrus LTP in awake rats, the F-actin content and intensity of drebrin A immunoreactivity are stably enhanced at medial perforant path synapses (Fukazawa et al., 2003). In adult drebrin A knockout mice, LTP in the hippocampal CA1 region and contextual fear memory are impaired (Kojima et al., 2016). Studies of chemically-induced LTP in cultured neurons support a two-stage model in which the exodus of short, drebrin A-decorated actin filaments from the spine head into dendritic shaft allows initial expansion of the F-actin network in spines, while reentry of drebrin stabilizes nascent filaments and supports long-term spine enlargement (Sekino et al., 2006; Mizui et al., 2014). Taken together current evidence implicates drebrin A in the fast transition between dynamic and stable states of actin in dendritic spines (Shirao and González-Billault, 2013; Grintsevich and Reisler, 2014). As a working hypothesis we propose that SUMOylated Arc contributes to the dynamic phase of drebrin A incorporation into, and stablization of, nascent actin filaments during LTP.

SUMOylation reactions are typically transient and involve only a small fraction of the total substrate pool at steady state (Tirard et al., 2012; Flotho and Melchior, 2013; Henley et al., 2014). Consistent with this, we show that only a fraction of the Arc protein in complex with drebrin A is SUMOylated. The presence of drebrin/SUMO-Arc complexes in the basal state (contralateral and naïve dentate gyrus) may represent a subpopulation of synapses undergoing long-term modification, in-line with the small subpopulations of granule cells expressing Arc mRNA in behaving animals.

Cofilin and drebrin A are both side-binding regulators of actin filaments. Cofilin severs actin filaments and promotes turnover whereas drebrin A stabilizes filaments. During LTP consolidation, Arc synthesis is required to maintain cofilin in a phosphorylated, inhibited state (Messaoudi et al., 2007). The observed lack of coimmunoprecipitation between Arc and cofilin in the present study indicates that Arc’s impact on cofilin activity is indirect. As cofilin and drebrin are known to compete for binding to filaments (Zhao et al., 2006; Grintsevich and Reisler, 2014), it is possible that Arc impacts cofilin phosphorylation indirectly by modulating drebrin A activity. The reciprocal coimmunoprecipitation between Arc and drebrin A indicates a close interaction in a common complex, but not necessarily direct binding. Arc interaction with drebrin A may require other factors.

Arc has predicted consensus SUMOylation sites at lysine 110 and lysine 286 (Bramham et al., 2010), and mutation of these sites blocks *in vitro* Arc SUMOylation in HEK cells (Craig et al., 2012). In cultured hippocampal neurons, viral overexpression of the Arc-KK SUMO mutant, but not wild-type Arc, prevents tetrodotoxin-induced synaptic scaling, indicating that Arc SUMOylation may be required for homeostatic scaling of AMPARs (Craig et al., 2012). Overexpression of Arc-KK does not affect GluA1 endocytosis, suggesting that Arc SUMOylation promotes the forward trafficking of GluA1 to the cell surface and thus favors homeostatic potentiation of synapses during chronic acitivity blockade (Craig and Henley, 2012; Craig et al., 2012). It is further noteworthy that drebrin A, which we have shown interacts with SUMOylated Arc, is implicated in the activity-dependent membrane insertion of AMPARs (Kato et al., 2012).

Although *in vitro* evidence implicates consensus lysine residues, the site(s) of SUMO conjugation on endogenous Arc have not been defined. SUMOylation commonly occurs on non-consensus lysines, whereas predicted consensus lysines are often not SUMOylated (Flotho and Melchior, 2013; Henley et al., 2014). In the study of Craig et al. (2012), 75 kDa and 120 kDa SUMO-reactive bands were immunoprecipitated from rat brain lysate using anti-Arc antibody, consistent with double and polySUMOylation. Using SUMO1 immunoprecipitation from rat dentate gyrus lysates, synaptoneurosomes and cytoskeletal fractions, we deteted a single high molecular weight Arc-immunoreactive species at 65 kDa, indicating single SUMO1 modification of Arc, as also seen by *in vitro* SUMOylation of immunoprecipitated Arc.

Arc is known to be ubiquitinated on lysine 268 and 269 by the E3 ligases Triad3A and Ube3a and targeted for proteasomal degradation (Rao et al., 2006; Greer et al., 2010; Soulé et al., 2012; Kühnle et al., 2013; Mabb et al., 2014). If SUMO and ubiquitin compete for modification of lysine residues on Arc, SUMOylation would be predicted to slow Arc degradation. However, Arc wildtype and Arc KK mutant have similar degradation rates when overexpressed in cultured hippocampal neurons (Craig et al., 2012), and likewise we saw rapid degradation of endogenous and SUMOylated Arc in the context of LTP.

Another salient finding of the present study was the presence of non-modified 50 kDa Arc in the SUMO1 and SUMO2/3 precipitate, demonstrating non-covalent interaction of Arc with SUMOylated proteins or with free SUMO. SUMO-interacting motifs (SIMs) typically consist of a hydrophobic core flanked by acidic residues (or phosphorylatable serine residues; Gareau and Lima, 2010). A major role of SIMs is to allow formation of specific SUMO-based interaction complexes, and many neuronal and synaptic proteins appear to contain SIMs (Feligioni et al., 2009; Wilkinson et al., 2010). Sequence alignment of Arc with known SIMs (RanBP2, Human PML and Daxx; Song et al., 2004; Lin et al., 2006) predicts a SIM-like motif (_317_**EEEEIIQYVV**) in the Arc C-terminal domain. However, it remains to be seen whether Arc binds SUMO directly. Recovery of unmodified Arc in the SUMO pellet was highly NEM-dependent, indicating that Arc interacts with SUMOylated proteins rather than to free SUMO. This interaction was detected in lysates and synaptoneurosomes but not in the cytoskeletal fraction, suggesting that non-covalent interaction of Arc with SUMOylated proteins occurs in the spine compartment but not in association with the actin cytoskeleton.

SUMOylation has recently been shown to inhibit or promote specific protein aggregation in neurons. The translational repressor, cytoplasmic polyadenylation binding protein (CPEB3), undergoes activity-dependent deSUMOylation resulting in aggregation of CPEB3 and enhanced translation (Drisaldi et al., 2015). In contrast, aggregation of α-synuclein in dopaminergic neurons of the substantia nigra is inhibited by SUMOylation (Krumova et al., 2011). Recent work showed that recombinant human Arc exists as a monomer but is capable of reversible self-oligomerization (Byers et al., 2015; Myrum et al., 2015). It will therefore be important to determine if SUMOylation impacts the oligomeric state of Arc.

Arc can be thought of as a flexible hub protein and organizer of neuronal plasticity through interaction with multiple protein partners in different subcellular compartments. The present works strongly implicates SUMOylation in the regulation of Arc localization and function in synaptic plasticity *in vivo*. Further work is needed to identify SUMO-directed protein-protien interactions and causal roles of Arc SUMOylation.

## Author Contributions

RRN and CRB conceived the study and designed the experiments. SP, AT, DP and LS performed *in vivo* electrophysiology and analysis. RRN, SP, DP, TK and KP performed biochemical experiments and analysis. GW co-supervised biochemical experiments done by KP. SP and CRB wrote the article with contributions from all authors. All authors approved the final version of the manuscript.

## Funding

Supported by The Research Council of Norway (grants 186115, 204861, 199355, 249951, 226026 to CRB), Polish-Norwegian Research Fund (Grant PNFR-96 to GW and CRB), and the Norwegian Financial Mechanism (Grant EMP128 to CRB). This work was funded by the EU Joint Programme—Neurodegenerative Disease Research (JPND) project, CircProt; JPND funding to CRB is supported by the Research Council of Norway.

## Conflict of Interest Statement

The authors declare that the research was conducted in the absence of any commercial or financial relationships that could be construed as a potential conflict of interest.

## Abbreviations

AMPA: α-Amino-3-hydroxy-5-methyl-4-isoxazolepropionic acid
Arc: activity-regulated cytoskeleton-associated protein
BDNF: brain-derived neurotrophic factor
HFS: high-frequency stimulation
LTP: long-term potentiation
PSD-95: postsynaptic density protein-95
SUMO: Small ubiquitin-like modifier.
