# Supplementary material for: Dynamic Arc SUMOylation and Selective Interaction with F-Actin-Binding Protein Drebrin A in LTP Consolidation In Vivo
Source: Front Synaptic Neurosci. 2017 May 10;9:8. doi: 10.3389/fnsyn.2017.00008 (PMC5426369; doi:10.3389/fnsyn.2017.00008)

Supplementary material

SUPPLEMENTAL FIGURE 1.

(A) Sample blot of SUMO1 immunoprecipitation, Arc immunoblot. (B) Sample blot of Arc immunoprecipitation, SUMO1 immunoblot. Bidirectional coimmunoprecipitation of dentate gyrus lysate samples detects a 65 kDa Arc and SUMO1 immunoreactive band that increases in intensity in the HFS-treated dentate gyrus.

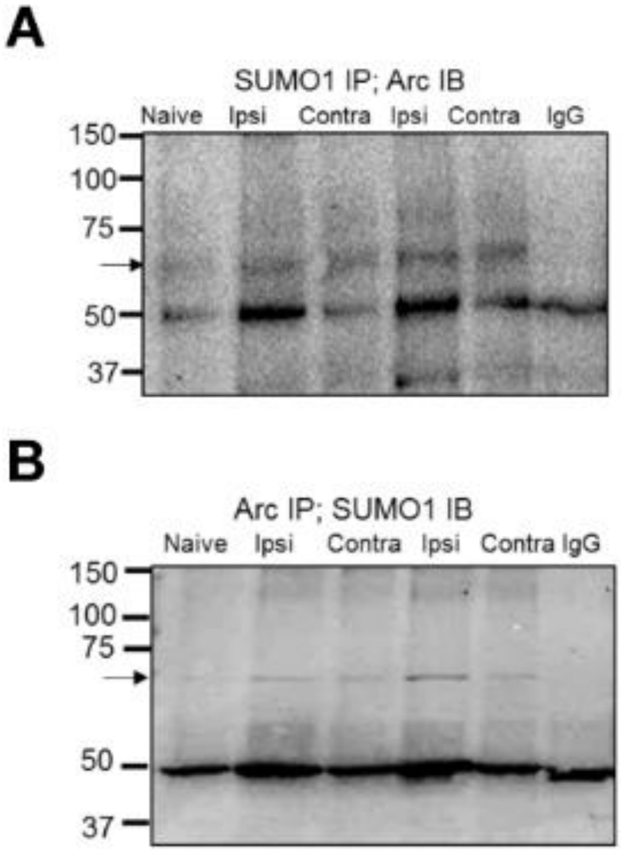

SUPPLEMENTAL FIGURE 2.

A-C. Coimmunoprecipitation of Arc binding partners. Arc was immunoprecipitated from dentate gyrus lysates obtained 1 hour post-HFS, and the resulting pellet was immunoblotted for candidate binding partners. **(A)** CaMKIIβ and CaMKIIα (**B**), PSD-95 (**C**) dynamin 2.
(D-F) Reverse immunoprecipitation. Non-modified 50 kDa Arc (but not 65 kDa SUMO1-ylated Arc) is detected in complex with immunoprecipitated CaMKIIα, CaMKIIβ, PSD-95, and dynamin 2. Representative blots based on at least three independent biological replicates (LTP experiments). Blots in each panel are from a single gel.

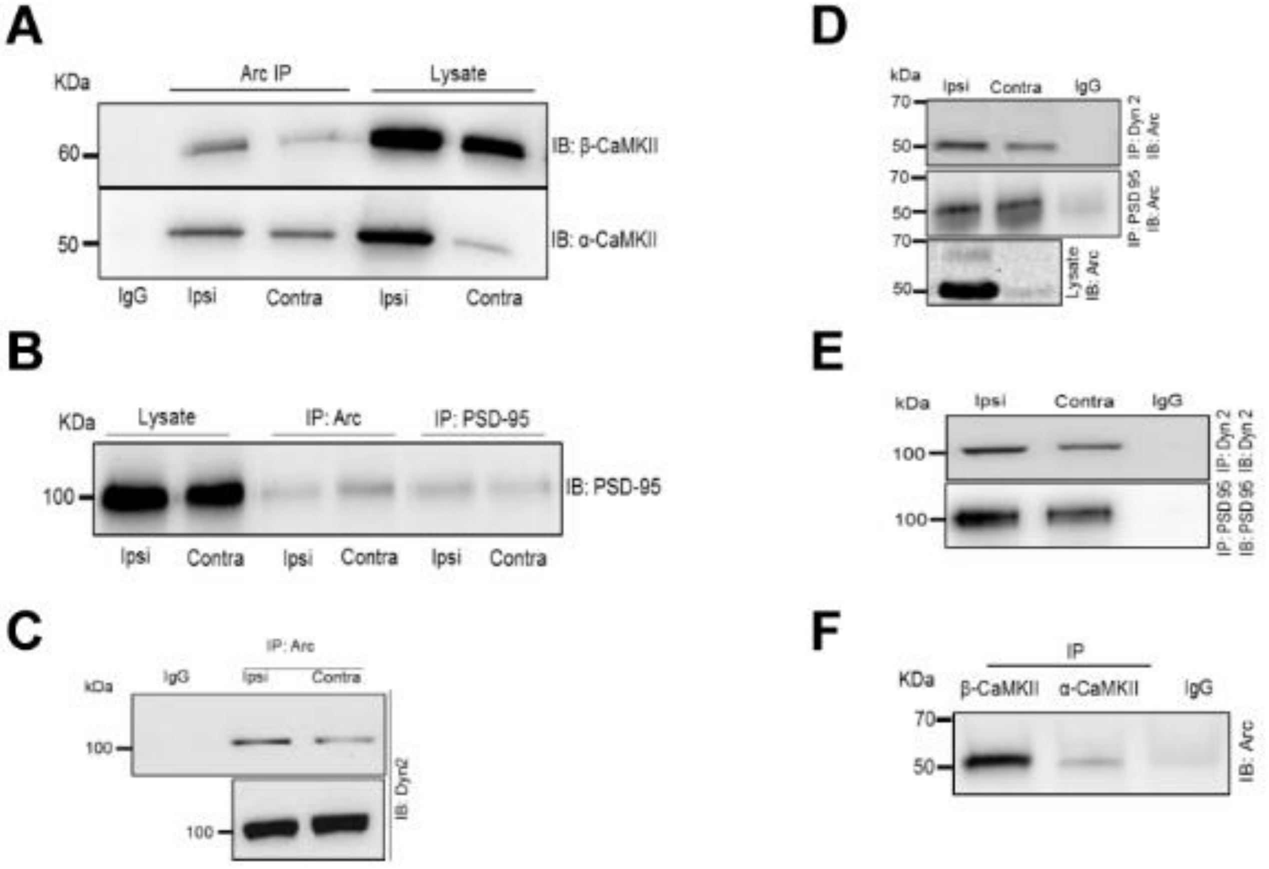

Supplement: Supplementary file 1 [file Image_1.pdf]
